# Seven lessons for financial incentivisation schemes in healthcare: a qualitative study of NHS Resolution’s Maternity Incentive Scheme

**DOI:** 10.1177/01410768261476118

**Published:** 2026-08-29

**Authors:** Graham Paul Martin, Gizdem Akdur, Jane O’Hara, Mary Dixon-Woods

**Affiliations:** University of Cambridge, Cambridge, UK

**Keywords:** Health policy, health service research, qualitative research, evidence based practice, obstetrics and gynaecology, patient safety

## Abstract

**Objectives:**

Avoidable harm and variations in quality have remained persistent features of health systems, but the track record of financial incentives in driving improvement is mixed. Based on an evaluation of NHS Resolution’s Maternity Incentive Scheme (MIS), which focuses on safety of maternity care in English NHS trusts, we identify generalisable principles to inform design of financial incentive schemes in healthcare.

**Design:**

Qualitative process evaluation based on interviews with those involved in the design and delivery of MIS.

**Setting:**

Organisations involved in running MIS and organisations participating in the scheme.

**Participants:**

Thirty-five individuals were interviewed, including 17 involved in the national-level administration of MIS and 18 with experience of MIS at local level.

**Main outcome measures:**

Not applicable.

**Results:**

We identified strengths, weaknesses and unintended consequences of the Scheme from the perspective of participants. MIS was seen by national-level stakeholders as having succeeded in elevating maternity safety in organisations’ priorities and consolidating disparate safety requirements into a unified framework. They saw MIS as a clinically credible and system-owned mechanism for prioritisation. However, local-level participants reported significant administrative burdens, opportunity costs and challenges in interpreting and implementing its requirements. The Scheme’s all-or-nothing reimbursement model, while reinforcing parity across the areas it covered, sometimes led to disproportionate focus on marginal issues and created perverse incentives when full compliance seemed unattainable. We identify seven key lessons for the design of incentive systems that might optimise their impact from the findings.

**Conclusions:**

Careful design of incentive schemes is vital to minimise unintended consequences. Lessons from MIS are relevant to broader healthcare system reform efforts and initiatives aiming to harness financial levers for quality improvement, such as those in the 10-Year Health Plan for England.

## Background

Preventable harm persists in healthcare systems worldwide,^1^ and the many efforts to improve safety and quality have a mixed track record.^2^ Approaches to improvement have been wide-ranging, from mandatory targets through to self-organising networks, each with strengths and weaknesses.^3^ Recently, the English National Health Service (NHS) has seen a resurgence of interest in the role of financial incentives in improving healthcare. The recent *10-Year Health Plan for England*, for example, calls for ‘a more consistent approach to using financial incentives to recognise and reward changes that deliver value and improve outcomes’, and includes proposals for financially rewarding and penalising provider organisations based on measures of effectiveness, integration and patient experience.^4^

Financial incentives have their own mixed history in supporting improvement. Their unintended consequences^5,6^ range from gaming (manipulation of data to look good)^7^ to effort substitution (focusing on the things being measured to the exclusion of others)^8,9^ and goal displacement (preferring activities that are most easily achieved).^10^ Consequently, evidence for the net effect of financial incentives on quality of care is equivocal,^11,12^ but it does indicate that system design – including choices about what to incentivise, how to measure it and the scope of incentivisation – is crucial. For example, incentives that capture the principal aims of a service and can be meaningfully and accurately measured may be more resistant to unintended consequences,^13^ while a limited set of incentives will offer more clarity on what is expected of organisations than a system that incentivises too much.^14,15^

One notable financial incentivisation initiative is the Maternity Incentive Scheme (MIS), launched in 2017, and operated by NHS Resolution.^16^ In a field characterised by long-standing safety concerns and large financial liabilities for the NHS,^17–19^ MIS aims to deliver safer maternity services and reduce incidence of brain injuries or other harm that can lead to negligence claims by financially rewarding compliance with 10 standardised ‘safety actions’. Each year, trusts that provide maternity services and are indemnified by NHS Resolution’s Clinical Negligence Scheme for Trusts (CNST) pay an additional 10% of the maternity element of their CNST premium. Trusts are then asked to declare their compliance with the 10 safety actions (Table 1), which were developed by NHS Resolution, the Chief Midwifery Officer and the National Clinical Director for Maternity in partnership with an advisory group of national agencies (Box 1). Trusts that declare compliance with all 10 safety actions receive a full reimbursement of their MIS contribution plus a share of unallocated funds. Those that declare less-than-full compliance do not receive their MIS contribution back, though they can bid for a smaller amount of discretionary capped funding to support maternity safety improvement activities.^16^

**Table 1.** MIS safety actions (Year 7).

No.	Safety action	Lead organisation
1	Are you using the National Perinatal Mortality Review Tool to review perinatal deaths to the required standard?	MBRRACE-UK
2	Are you submitting data to the Maternity Services Data Set to the required standard?	NHS England
3	Can you demonstrate that you have transitional care services in place to minimise separation of mothers and their babies and to support the recommendations made in the Avoiding Term Admissions into Neonatal units programme?	NHS England
4	Can you demonstrate an effective system of medical workforce planning to the required standard?	RCOG
5	Can you demonstrate an effective system of midwifery workforce planning to the required standard?	RCM
6	Can you demonstrate that you are on track to compliance with all elements of the Saving Babies’ Lives Care Bundle version 3?	NHS England
7	Can you demonstrate that you listen to women, parents and families using maternity and neonatal services and coproduce services with users?	NHS England
8	Can you evidence three (specified) elements of local training plans and ‘in-house’, one day multiprofessional training?	RCOG and NHS England
9	Can you demonstrate that there are robust processes in place to provide assurance to the Board on maternity and neonatal safety and quality issues?	NHS England
10	Have you reported 100% of qualifying cases to the Maternity and Newborn Safety Investigations (MNSI) programme and to NHS Resolution’s Early Notification Scheme?	NHS England & MNSI

Source: NHS Resolution.^16^

**Box 1.** MIS collaborative advisory group members.

• NHS Resolution
• NHS England
• Royal College of Obstetricians and Gynaecologists (RCOG)
• Royal College of Midwives (RCM)
• Mothers and Babies: Reducing Risk through Audits and Confidential Enquiries (MBRRACE-UK)
• Royal College of Anaesthetists
• Neonatal Clinical Reference Group
• Care Quality Commission (CQC)
• Maternity and Newborn Safety Investigation (MNSI) programme (previously Healthcare Safety Investigation Branch)

Source: NHS Resolution.^16^

Maternity care has faced an overwhelming number of competing recommendations and requirements that risk diluting organisational attention.^18,20^ One benefit of MIS, therefore, is that it may offer some clarity on what trusts should prioritise to improve maternity safety. An internal evaluation of MIS reported ‘demonstrable success in driving improvements’ in safety,^21^ and there are some quantitative indications that the Scheme has succeeded in improving compliance. In its first year (2017–18), 75 of 132 trusts (57%) certified as achieving all 10 actions.^21^ By its sixth year (2024–25), this had improved to 102 of 120 trusts (85%).^22^ In this paper, we report a qualitative evaluation of MIS that examined both the organisation of the Scheme at national level and the response in provider organisations and other local-level bodies, identifying strengths, weaknesses and unintended consequences of an incentive initiative with promising results.

## Methods

Our evaluation involved qualitative interviews with two sets of stakeholders between November 2024 and February 2025. Firstly, we interviewed those involved in the administration of MIS and members of its advisory group of national-level stakeholders. These individuals were identified and approached by NHS Resolution and are labelled ‘National’ in our findings. Secondly, we interviewed those in NHS trusts and other organisations (e.g. integrated care systems and local maternity and neonatal systems) involved in responding to the MIS locally, for example by undertaking audits, ensuring compliance and signing off declarations. These individuals, labelled ‘Local’ in our findings, were recruited using a range of approaches, including publicising the study on the online FutureNHS platform, direct approaches by NHS Resolution to individuals in organisations with varying levels of current and past compliance and ‘snowball’ sampling, whereby interviewees suggested further potential participants.

Based on the principles of information power,^23^ we anticipated a total sample of 30–35 participants would be sufficient. Specifically, we anticipated that the well-defined nature of the inquiry, the breadth, depth and complementarity of the experiences of the two groups of participants, and the existing body of research and theory relating to incentivisation meant that a sample of this size, which is within the typical range for a focused qualitative study of this kind,^24^ would be adequate. We kept sample size under review as data collection and analysis proceeded.

Participants received an information sheet, were given the opportunity to ask questions and provided written consent prior to interviews. Interviews lasted 52 min on average. They were guided by topic guides tailored to the two groups, covering for example how safety actions were selected for the scheme (national-level participants), the activity involved in formulating a MIS declaration (local-level participants) and the relationship between safety actions and the most pressing issues in maternity safety (both groups). All interviews were audio-recorded and fully transcribed prior to analysis.

Analysis was based on the framework approach,^25^ and involved review of transcripts and coding to themes identified prospectively (based on the evaluation’s objectives and the authors’ knowledge of relevant literatures) and inductively (themes arising from the data that had not been anticipated by the authors). Following initial coding, further cross-transcript review based on the initial coding produced further refinements to the coding framework and was used to produce the analysis. Based on further discussion within the research team, we identified key lessons arising from our analysis – and particularly from the gaps we identified between how the Scheme was intended to operate and how it was experienced on the ground – likely to be transferable to other incentive schemes.

Based on the Health Research Authority decision tool, the study was deemed service evaluation and was thus ineligible for research ethics committee review. However, informed consent was obtained from all participants.

## Results

We interviewed 35 participants: 17 with involvement in the national-level administration of MIS and 18 with experience of local organisations’ submissions to MIS (principally provider trusts), from all 7 English NHS regions. National-level participants typically had trust-level experience of collating submissions. Considering two key components of MIS – firstly, its attempt to provide clarity for organisations on what to prioritise among a surfeit of competing pressures; secondly, the use of incentivisation to ensure focus in organisational action – we compare the Scheme’s intentions with how it was experienced ‘on the ground’. In the process, we derive seven lessons for incentive schemes that we set out in more detail in Box 2 and in the discussion section.

**Box 2.** Seven lessons for the design and implementation of incentives schemes in healthcare.

**1. Prioritisation alone may not untangle ‘priority thickets’**
Providing clarity of purpose and cutting through the priority thickets^15^ that dilute organisational attention is vital. However, in a complex and dynamic system, it may be beyond the ability of any single agency to rationalise the ask of organisations, even in collaboration with other influential bodies.
**2. Be alert to the implications of a changing environment**
Clinical developments, shifts in population need and changes in organisational performance may have implications for where incentives might yield the greatest impact. Changes in the regulatory environment, such as new agencies, initiatives and mandates, may alter the effectiveness of incentivisation and the competing demands for organisational attention.
**3. The administrative burden of incentivisation can be substantial and the opportunity costs are real**
The work of interpreting requirements, setting up measurement systems, undertaking audits and improving quality is very substantial and often falls at the sharp end of organisations, potentially displacing other valuable work – for example by consuming professional time. Clearly specified requirements, aligned as much as possible with existing reporting systems, are vital. The costs of reporting need to be fully accounted for, especially when introducing new requirements.
**4. Balance the competing needs for stability and evolution**
Stability and consistency of purpose and objectives may help to mitigate the administrative burden of incentive schemes, but responding to the changing environment is vital to their impact and legitimacy. If it is necessary to incentivise new areas of activity, consider whether existing incentives might be simplified or reduced.
**5. Think carefully about what you want board engagement to achieve**
Financial incentives appear to be effective in capturing organisational attention at senior levels, but how this translates into board behaviour may vary – and with it the impact on behaviour at the level of clinical services. Theories of change for incentive systems should account for intra-organisational as well as system-level mechanisms.
**6. Unintended consequences are not the only threat to integrity of incentivisation**
While the potential unintended consequences of incentive schemes are well documented, even a scheme working as intended may have downsides – for example, by distorting the level of attention given to interpreting, measuring or achieving incentivised goals.
**7. Consider the risk of exacerbating inequalities in performance**
Incentive schemes carry the risk of increasing inequalities between high-performing and low-performing organisations, both directly, through resource allocation that rewards high-performers, and indirectly, if organisations that anticipate low likelihood of success become disengaged.

### Prioritisation: a mandate for action?

National-level participants identified key achievements of MIS as, first, consolidating existing requirements that units were already expected to meet into a single national set and, second, providing powerful incentives for compliance. They proposed that MIS’s 10 safety actions offered valuable clarity to organisations on what to prioritise, underpinned by incentives that would command action.

MIS is not creating actions, it’s not designed to say, ‘We, NHSR, think you should be doing the following’. What it’s designed to do is provide the financial lever to incentivise things which maternity units are already being asked to deliver by, for example, NHS England. (National10)

They also suggested that the process for selecting the 10 actions, in consultation with an advisory group including major national bodies, helped to secure legitimacy for the Scheme and ensure that it integrated, rather than competed with, the various top-down demands being made of organisations. The clinical credibility of the advisory group’s membership, national-level participants argued, meant that it appealed to the professional sensibilities as well as the financial interests of those in provider organisations.

[MIS] is led from within the system, so it is clinically owned, it is collectively governed. (National13)

Within the set of 10 actions, national-level participants were clear that there was no hierarchy. The 10 had been chosen based on their likely impact, as agreed by the advisory group; they were also seen as complementary in the way they addressed different but vital aspects of the maternity care pathway. The rules of the Scheme – particularly its ‘all-or-nothing’ approach, whereby trusts could only obtain reimbursement of their payment by declaring compliance with all 10 safety actions – were seen to reinforce this message.

At local level, participants had more mixed views of the importance of the 10 safety actions. Many reported that huge amounts of work were involved in interpreting the actions, securing the coordinated effort needed to provide internal assurance on compliance and collating and reporting data through local governance structures. In contrast to national-level participants, they generally did not see MIS as providing a single, definitive mandate that rationalised all expectations of their services. On the contrary, they reported that they were still obliged to meet the requirements of other bodies and regulators, new maternity safety initiatives continued to be launched, and multiple recommendations for improvement continued to emerge from many different sources (lessons 1 and 2). They expressed concerns about failures to align timelines and reporting requirements of the many bodies to which they were accountable.

We’ve got lots of disconnected compliance and quality and safety stuff. [. . .] Over 380 lines of compliance and that’s without all the things that don’t have those numerical bounds or time bounds around it. So, we’ve also got the Three-year Delivery Plan, we should also be working on Ockenden. [. . .]So there is no one person at the top of NHS England perinatal going, ‘OK, this all sounds like it’s a bit much’. (Local16)

Beyond these additional demands, local-level participants reported significant administrative burden associated with the MIS itself (lesson 3). The headline of 10 safety actions, they noted, masked many more ‘sub-actions’ – peaking at over 170 in the fifth year of the Scheme – that consumed organisational attention and professional and managerial effort.

MIS is too quick, so the focus is on continually collecting data. [. . .] I think you do change targets with very little lead-in time and that’s endemic of the NHS as a whole anyway, that everything is very short term. (Local15)

Those involved in the Scheme’s design acknowledged the temptation to maximise the value gained from a seemingly effective scheme – ‘everyone, and myself included, sneak extra ones in within their safety action’ (National03) – and noted a concerted effort to reduce the administrative burden in Year 6 of the Scheme (lesson 4).

Local-level participants also reported that the opportunity costs of MIS were significant. A particular concern was the diversion of experienced staff from direct patient care to work on MIS administration. Some local-level participants doubted whether devoting scarce professional time to such activities was aligned with the interests of patient safety or whether it addressed the most pressing needs of the populations served.

[We’re] taking an experienced midwife [. . .] out of the pool from patient care. (Local14)

It’s just an industry of producing information for different people rather than actually doing the things that we’re supposed to do to keep things safe. We spend our life writing things down for different people. (Local13)

### Incentivisation: inducing the right behaviours?

National-level participants largely agreed that financial incentivisation had succeeded in raising the profile of maternity safety among trust boards and increasing the interest they took in maternity safety: MIS, as National05 put it, was a ‘fantastic lever to get the board’s attention’. They described the potentially high impact of failing to recoup MIS payments, since organisations could lose very significant (six- or seven-digit) sums, depending on the size of their premiums.

Local-level participants concurred that MIS had raised maternity care up the organisational agenda. However, they were more sceptical about whether increased board recognition had consistently translated into effective support for maternity and neonatal services in delivering the safety actions (lesson 5). They also questioned the validity of MIS compliance as a proxy for safety, in part because some saw it as a triumph of reporting over practice, particularly as interpretation of points of detail could vary.

There is something for me about the complexity of each individual point because it’s really difficult to hold and if you’re lucky enough to have somebody leading on it, you’re relying on their interpretation of what that means. [. . .] I certainly know that our directors of midwifery [have] tripped on a detail or something that has been out of their control [that] has then caused them to fail. (Local16)

Some expressed concerns about an approach that, by design, penalised trusts for non-compliance, with potential to take significant amounts of money from organisations for shortcomings not wholly within their control (e.g. workforce planning that might be derailed by local labour markets). They suggested that a combination of reliance on self-declaration by hospital trusts (with oversight from local commissioners) and the penalties for non-compliance risked producing results that, even if not deliberately deceitful, might be susceptible to optimism bias.

From a trust executive financial point of view, I think there is a lot of pressure on people to declare full compliance. [. . .] Please don’t think that I think that people lie. But I think their interpretation of the safety actions is a little loose at times. So therefore I don’t think that’s necessarily a guarantee that maternity units are safe. (Local07)

Local-level participants identified another consequence of MIS’s all-or-nothing approach to incentivisation. While it successfully conveyed the parity of the 10 actions, including those (such as co-production with maternity service users) that might otherwise have been deferred, it also meant that where achieving one action might make or break full compliance, that action could assume disproportionate importance (lesson 6).

In our trust we had to update 11 guidelines [to comply with safety action 6]. Obviously, you can’t just update a guideline within a month. You’ve got to have a specific lead leading on it. It takes time. It then goes through the governance process, drafting, comments. [. . .] Depending on your trust’s internal process, it may take a month once it’s been commented on and drafted to be approved. But then if it’s got medicines in it, it will take extra—even longer. So, we had to prioritise the guidelines that were purely for [safety action 6] over all our other guidelines that were overdue. Which still could have been a safety issue. (Local10)

Conversely, where trusts saw little prospect of achieving the bar of full compliance, they could be tempted simply to write off their MIS contribution and focus their interest elsewhere instead – to the detriment of maternity safety (lesson 7).

It was more to do with the amount of work that was going to be taken up. So they felt that actually, the gap between what was going to be needed in terms of the amount of work, and therefore the amount of resource that they were going to have to put in to achieving it, wasn’t going to be worth it in terms of the rebate, and also it wasn’t going to improve their safety, was how they felt, and that they would rather use that resource in other ways for their organisation. (National17)

## Discussion

Our study of 35 stakeholders involved in NHS Resolution’s Maternity Incentive Scheme offers important insights into the operation of a financial incentive scheme for patient safety in the English NHS that, on the face of it, has secured major improvements over time. National-level participants perceived that the Scheme rationalised and consolidated safety requirements, offered clarity on what should be prioritised, elevated maternity care at board level and secured better compliance. Local-level participants largely agreed that MIS had raised the profile of maternity services at board level, but were less convinced that it had driven authentic improvement at service level. In a complex and changing landscape of overlapping recommendations, standards and oversight bodies, MIS was experienced at the sharp end of local care as doing little to reduce ‘priority thickets’,^15^ and instead was often seen as an extra quasi-regulatory demand. It was perceived as generating significant administrative burdens that could detract from safety by diverting staff from the shop floor. The all-or-nothing approach to incentivisation, which sought to ensure that all 10 safety actions were treated with equal gravity, was seen as vulnerable to unwanted consequences, from disproportionate focus on seemingly marginal issues to a tactical decision to abandon work towards compliance if it looked too remote a prospect.

Based on our analysis, we identify seven lessons that might inform the design and administration incentivisation schemes more broadly, subject to further evaluation (Box 2).

### Strengths and limitations

This study is the first external evaluation to examine the implementation of MIS. It uses robust qualitative methods that engaged the views of those running the Scheme and those whose day-to-day work is affected by it. Approaches to recruitment sought to ensure that those with positive and negative views of the Scheme would participate, but the fact that the study was commissioned by NHS Resolution might have deterred some possible participants. While qualitative data are valuable in offering insight into the mechanisms through which interventions operate, they cannot evaluate effectiveness, and reliance on interview accounts means that our findings may be subject to social-desirability bias^26^ and related limitations.

### Implications for policy

Our study suggests that MIS is a potentially effective but imperfect tool within a broader system of maternity safety governance and patient safety performance management. It also offers valuable lessons for other similar initiatives (Box 2), including those envisioned in current NHS reform plans – though these principles would benefit from further testing in new contexts.

The design of MIS sought explicitly to address some of the fallibilities of incentive systems: for example, by seeking national-level agreement on a parsimonious set of safety actions, it sought to mitigate the risk of effort substitution. The all-or-nothing approach to reimbursement might be expected to reduce the risks of goal displacement.^8,10^ But these features of the Scheme produced their own effects that, if not exactly unintended, could threaten its fundamental aims – as well as its legitimacy in the eyes of stakeholders. Where participants saw organisations agonising over seemingly marginal issues that could make or break compliance, for example, they were unconvinced that this was the most effective way of improving safety. Our analysis also identified some familiar unintended consequences of incentive schemes, including the temptation to prioritise ‘paper compliance’ over substantive change or ‘hitting the target but missing the point’.^7,27^ However, reports of intentional gaming – so often a concern in previous iterations of incentives schemes – did not feature in our interviews. Rather, participants suggested that ambiguity in the detail of the specific activities required under each safety action, and a tendency towards optimism bias associated with self-reporting, might compromise the validity of MIS declarations.

In a complex, politicised and dynamic system such as maternity care, MIS faced challenges in balancing consistency of purpose with responsiveness to change. Local-level participants found year-to-year changes in specifications of safety actions and sub-actions frustrating. But they also noted that if it was to maintain its claim to rationalising national priorities, MIS needed to adapt and incorporate emergent national imperatives, triggered for example by new investigations and government responses to them. National-level participants noted the need for evolution in response to a changing political, clinical and demographic environment, but recognised the imperative to contain MIS requirements and avoid the expansionist tendencies of its early years.

Our findings, and their implications for incentive system design (Box 2), will be of interest to policymakers as they seek to harness the power of incentivisation while avoiding its well documented pitfalls. The *10-Year Health Plan*, for example, anticipates greater use of incentives at individual and organisational level, but notes the need for parsimony, stating that ‘lack of clarity in the system about roles and purpose’ must ‘be fixed by establishing clear priorities’ and ‘mandating fewer targets’.^4^ The Plan’s proposals for ‘Patient Power Payments’ bear a striking resemblance to some aspects of MIS, involving the holding back of a proportion of funding to providers contingent on patient satisfaction, with the money being used elsewhere in the NHS if organisations fall short of the standard. Our study shows that care in the design of initiatives like this will be vital if their positive impact is to be optimised and their potential downsides mitigated.

A further recent government-commissioned review has called for rationalisation of national agencies with an interest in patient safety, and a key role for the NHS’s National Quality Board to ‘prioritise new and existing recommendations’ made to NHS organisations and ‘ensure that unfunded mandates (such as those arising from reports, reviews, inquiries, investigations, guidance and activities of various bodies) are not imposed on providers without due diligence’.^28^ Our study suggests that while such an initiative is timely, and likely to be welcomed by organisations that find themselves overwhelmed by top-down demands, it will not be easy – especially given a system so subject to shifts in evidence, clinical need and political attention.

## Conclusion

This study offers valuable learning about the design and operation of financial incentive schemes. While MIS’s success in raising the profile of maternity safety was largely agreed and the impacts on reported compliance were evident, the impact on practice at service level was more ambiguous, so the Scheme’s net effect on safety was more questionable. These findings also show that an incentive scheme that is functioning exactly as intended can still result in sub-optimal outcomes, quite apart from any unintended consequences. The implications of our findings, summarised in seven lessons for those designing and implementing schemes of this kind, will be of interest to policymakers in the NHS and beyond as they seek to channel the power of incentivisation.

## Declarations

**Ethical approval:** Based on the Health Research Authority decision tool, the study was deemed service evaluation and was therefore ineligible for research ethics committee review.

**Guarantor:** GM.

**Contributorship:** GM: conceptualisation; methodology; formal analysis; investigation; writing – original draft; writing – review & editing; supervision; funding acquisition. GA: methodology; formal analysis; investigation; data curation; writing – review & editing; project administration. JO: conceptualisation; methodology; writing – review & editing; funding acquisition. MD-W: conceptualisation; methodology; writing – review & editing; funding acquisition.

**Provenance:** Not commissioned; peer-reviewed by Julie Morris.

**Use of generative AI:** No generative AI was used during the preparation of this manuscript.
